# Supplementary material for: Metabolic profiles among COPD and controls in the CanCOLD population-based cohort
Source: PLoS One. 2020 Apr 10;15(4):e0231072. doi: 10.1371/journal.pone.0231072 (PMC7147771; doi:10.1371/journal.pone.0231072)
Supplement: S1 Table — (DOCX) [file pone.0231072.s001.docx]

**Table S1** Multivariate logistic regression on Triglycerides > 1.5 mmol/L

|  | **OR** **(95%CI)** | | **p - value** | |
| --- | --- | --- | --- | --- |
| **COPD** | | 1.36 (0.73 ; 2.55) |  | 0.330 |
| **Age (years)** | |  |  | 0.621 |
| <60 | | Ref. |  |  |
| 60-65 | | 0.95 (0.41 ; 2.22) | 0.903 |  |
| 66-70 | | 1.15 (0.50 ; 2.67) | 0.739 |  |
| >70 | | 0.68 (0.29 ; 1.61) | 0.378 |  |
| **Sex (men)** | | **2.18 (1.04 ; 4.57)** |  | **0.039** |
| **BMI (Kg/m^2^)** | |  |  | **0.011** |
| <23.6 | | Ref. |  |  |
| 23.6-26.5 | | 1.03 (0.40 ; 2.64) | 0.957 |  |
| 26.6-29.3 | | 2.28 (0.89 ; 5.81) | 0.084 |  |
| >29.3 | | **3.91 (1.47 ; 10.41)** | **0.006** |  |
| **Waist/Hip ratio** | |  |  | **0.002** |
| <0.87 | | Ref. |  |  |
| 0.87-0.93 | | **6.08 (2.19 ; 16.90)** | **0.001** |  |
| 0.94-0.99 | | **6.73 (2.41 ; 18.78)** | **<0.001** |  |
| >0.99 | | **5.02 (1.54 ; 16.38)** | **0.008** |  |
| **Tobacco status** | |  |  | 0.933 |
| Never smoker | | Ref. |  |  |
| Former smoker | | 1.24 (0.63 ; 2.46) | 0.763 |  |
| Current smoker | | 1.09 (0.45 ; 2.64) | 0.970 |  |
| **Hypolipemic treatment** | | 0.84 (0.44 ; 1.61) |  | 0.487 |
| **Inhaled corticosteroid treatment** | | 0.95 (0.42 ; 2.18) |  | 0.885 |

Significant p-values and OR are shown in bold. COPD: chronic obstructive pulmonary disease; BMI: body mass index. Ref.: reference category. Cox-Snell Model R^2^ = 0.17
