# Supplementary material for: Metabolic profiles among COPD and controls in the CanCOLD population-based cohort
Source: PLoS One. 2020 Apr 10;15(4):e0231072. doi: 10.1371/journal.pone.0231072 (PMC7147771; doi:10.1371/journal.pone.0231072)
Supplement: S2 Table — (DOCX) [file pone.0231072.s002.docx]

**Table S2** Multivariate logistic regression on Triglycerides > 1.5 mmol/L, **COPD 2+ only**

|  | **OR** **(95%CI)** | | **p - value** | |
| --- | --- | --- | --- | --- |
| **COPD 2+** | | 1.26 (0.55 ; 2.92) |  | 0.587 |
| **Age (years)** | |  |  | 0.560 |
| <60 | | Ref. |  |  |
| 60-65 | | 1.02 (0.37 ; 2.80) | 0.969 |  |
| 66-70 | | 1.16 (0.43 ; 3.10) | 0.775 |  |
| >70 | | 0.58 (0.22 ; 1.53) | 0.316 |  |
| **Sex (men)** | | 0.58 (0.20 ; 1.70) |  | 0.076 |
| **BMI (Kg/m^2^)** | |  |  | 0.062 |
| <23.6 | | Ref. |  |  |
| 23.6-26.5 | | 0.74 (0.23 ; 2.40) | 0.618 |  |
| 26.6-29.3 | | 1.38 (0.44 ; 4.36) | 0.586 |  |
| >29.3 | | 3.03 (0.94 ; 9.79) | 0.065 |  |
| **Waist/Hip ratio** | |  |  | **0.003** |
| <0.87 | | Ref. |  |  |
| 0.87-0.93 | | **6.84 (2.05 ; 22.86)** | **0.002** |  |
| 0.94-0.99 | | **9.78 (2.88 ; 33.23)** | **<0.001** |  |
| >0.99 | | **7.86 (1.90 ; 32.61)** | **0.005** |  |
| **Tobacco status** | |  |  | 1.000 |
| Never smoker | | Ref. |  |  |
| Former smoker | | 1.00 (0.42 ; 2.35) | 0.997 |  |
| Current smoker | | 1.01 (0.32 ; 3.23) | 0.988 |  |
| **Hypolipemic treatment** | | 0.94 (0.42 ; 2.10) |  | 0.880 |
| **Inhaled corticosteroid treatment** | | 1.19 (0.40 ; 3.59) |  | 0.756 |

Significant p-values and OR are shown in bold. COPD: chronic obstructive pulmonary disease; COPD2+: only GOLD stage 2 and 3 are compared with controls; BMI: body mass index; Ref.: reference category. Cox-Snell Model R^2^ = 0.20
