# Supplementary material for: Metabolic profiles among COPD and controls in the CanCOLD population-based cohort
Source: PLoS One. 2020 Apr 10;15(4):e0231072. doi: 10.1371/journal.pone.0231072 (PMC7147771; doi:10.1371/journal.pone.0231072)
Supplement: S3 Table — (DOCX) [file pone.0231072.s003.docx]

**Table S3** Multivariate logistic regression on TC/HDL > 4

|  | **OR** **(95%CI)** | **p - value** | |
| --- | --- | --- | --- |
| **COPD** | 0.72 (0.36 ; 1.47) |  | 0.367 |
| **Age (years)** |  |  | 0.162 |
| <60 | Ref. |  |  |
| 60-65 | 0.90 (0.37 ; 2.19) | 0.820 |  |
| 66-70 | 1.13 (0.46 ; 2.80) | 0.792 |  |
| >70 | 0.39 (0.14 ; 1.05) | 0.062 |  |
| **Sex (men)** | 0.51 (0.22 ; 1.18) |  | 0.115 |
| **BMI (Kg/m^2^)** |  |  | **0.002** |
| <23.6 | Ref. |  |  |
| 23.6-26.5 | 0.99 (0.34 ; 2.87) | 0.990 |  |
| 26.6-29.3 | **3.96 (1.35 ; 11.59)** | **0.012** |  |
| >29.3 | **6.13 (1.96 ; 19.22)** | **0.002** |  |
| **Waist/Hip ratio** |  |  | **0.010** |
| <0.87 | Ref. |  |  |
| 0.87-0.93 | 2.66 (0.90 ; 7.90) | 0.078 |  |
| 0.94-0.99 | **5.83 (2.00 ; 16.95)** | **0.001** |  |
| >0.99 | 2.66 (0.76 ; 9.37) | 0.127 |  |
| **Tobacco status** |  |  | 0.132 |
| Never smoker | Ref. |  |  |
| Former smoker | 0.59 (0.27 ; 1.28) | 0.180 |  |
| Current smoker | 1.44 (0.52 ; 4.01) | 0.483 |  |
| **Hypolipemic treatment** | **0.17 (0.07 ; 0.42)** |  | **<0.001** |
| **Inhaled corticosteroid treatment** | 0.45 (0.14 ; 1.37) |  | 0.159 |

Significant p-values and OR are shown in bold. TC: Total cholesterol; HDL: High density Lipoprotein; COPD: chronic obstructive pulmonary disease; BMI: body mass index. Ref.: reference category. Cox-Snell Model R^2^ = 0.21
