# Supplementary material for: Metabolic profiles among COPD and controls in the CanCOLD population-based cohort
Source: PLoS One. 2020 Apr 10;15(4):e0231072. doi: 10.1371/journal.pone.0231072 (PMC7147771; doi:10.1371/journal.pone.0231072)
Supplement: S4 Table — (DOCX) [file pone.0231072.s004.docx]

**Table S4** Multivariate logistic regression on TC/HDL > 4, **COPD 2+ only**

|  | **OR** **(95%CI)** | | **p - value** | |
| --- | --- | --- | --- | --- |
| **COPD 2+** | | 0.51 (0.19 ; 1.37) |  | 0.179 |
| **Age (years)** | |  |  | 0.104 |
| <60 | | Ref. |  |  |
| 60-65 | | 0.87 (0.31 ; 2.43) | 0.783 |  |
| 66-70 | | 0.77 (0.28 ; 2.18) | 0.628 |  |
| >70 | | **0.21 (0.06 ; 0.78)** | **0.020** |  |
| **Sex (men)** | | 0.71 (0.27 ; 1.88) |  | 0.484 |
| **BMI (Kg/m^2^)** | |  |  | **0.014** |
| <23.6 | | Ref. |  |  |
| 23.6-26.5 | | 0.58 (0.15 ; 2.23) | 0.426 |  |
| 26.6-29.3 | | 3.16 (0.92 ; 10.88) | 0.069 |  |
| >29.3 | | **4.80 (1.27 ; 18.12)** | **0.021** |  |
| **Waist/Hip ratio** | |  |  | 0.083 |
| <0.87 | | Ref. |  |  |
| 0.87-0.93 | | 2.86 (0.84 ; 9.76) | 0.093 |  |
| 0.94-0.99 | | **4.99 (1.46 ; 17.06)** | **0.010** |  |
| >0.99 | | 3.026 (0.69 ; 13.22) | 0.141 |  |
| **Tobacco status** | |  |  | 0.576 |
| Never smoker | | Ref. |  |  |
| Former smoker | | 0.62 (0.25 ; 1.53) | 0.299 |  |
| Current smoker | | 0.82 (0.23 ; 2.89) | 0.755 |  |
| **Hypolipemic treatment** | | **0.25 (0.09 ; 0.71)** |  | **0.009** |
| **Inhaled corticosteroid treatment** | | 0.71 (0.19 ; 2.69) |  | 0.609 |

Significant p-values and OR are shown in bold. TC: Total cholesterol; HDL: High density Lipoprotein; COPD: chronic obstructive pulmonary disease; COPD2+: only GOLD stage 2 and 3 are compared with controls; BMI: body mass index. Ref.: reference category. Cox-Snell Model R^2^ = 0.20
