# Supplementary material for: Metabolic profiles among COPD and controls in the CanCOLD population-based cohort
Source: PLoS One. 2020 Apr 10;15(4):e0231072. doi: 10.1371/journal.pone.0231072 (PMC7147771; doi:10.1371/journal.pone.0231072)
Supplement: S5 Table — (DOCX) [file pone.0231072.s005.docx]

**Table S5** Multivariate logistic regression on HOMA-IR > 3

|  | **OR** **(95%CI)** | | **p - value** | |
| --- | --- | --- | --- | --- |
| **COPD** | | 0.61 (0.32 ; 1.17) |  | 0.137 |
| **Age (years)** | |  |  | 0.452 |
| <60 | | Ref. |  |  |
| 60-65 | | 2.10 (0.86 ; 514) | 0.105 |  |
| 66-70 | | 1.45 (0.61 ; 3.43) | 0.396 |  |
| >70 | | 1.51 (0.65 ; 3.51) | 0.398 |  |
| **Sex (men)** | | 1.47 (0.60 ; 3.61) |  | 0.619 |
| **BMI (Kg/m^2^)** | |  |  | **<0.001** |
| <23.6 | | Ref. |  |  |
| 23.6-26.5 | | 1.95 (0.77 ; 4.95) | 0.158 |  |
| 26.6-29.3 | | **3.47 (1.36 ; 8.90)** | **0.009** |  |
| >29.3 | | **15.28 (5.40 ; 43.23)** | **<0.001** |  |
| **Waist/Hip ratio** | |  |  | 0.348 |
| <0.87 | | Ref. |  |  |
| 0.87-0.93 | | 1.50 (0.60 ; 3.74) | 0.384 |  |
| 0.94-0.99 | | 1.03 (0.40 ; 2.65) | 0.957 |  |
| >0.99 | | 2.18 (0.73 ; 6.54) | 0.163 |  |
| **Tobacco status** | |  |  | 0.347 |
| Never smoker | | Ref. |  |  |
| Former smoker | | 1.18 (0.58 ; 2.39) | 0.651 |  |
| Current smoker | | 0.59 (0.22 ; 1.63) | 0.310 |  |
| **Hypoglycaemic treatment** | | 2.15 (0.59 ; 7.84) |  | 0.245 |
| **Inhaled corticosteroid treatment** | | 2.60 (0.98 ; 6.87) |  | 0.055 |

Significant p-values and OR are shown in bold. HOMA-IR: Homeostasis model assessment of insulin resistance; COPD: chronic obstructive pulmonary disease; BMI: body mass index. Ref.: reference category. Cox-Snell Model R^2^ = 0.28
