# Supplementary material for: Metabolic profiles among COPD and controls in the CanCOLD population-based cohort
Source: PLoS One. 2020 Apr 10;15(4):e0231072. doi: 10.1371/journal.pone.0231072 (PMC7147771; doi:10.1371/journal.pone.0231072)
Supplement: S6 Table — (DOCX) [file pone.0231072.s006.docx]

**Table S6** Multivariate logistic regression on HOMA-IR > 3, **COPD 2+ only**

|  | **OR** **(95%CI)** | | **p - value** | |
| --- | --- | --- | --- | --- |
| **COPD 2+** | | 0.70 (0.29 ; 1.67) |  | 0.421 |
| **Age (years)** | |  |  | 0.530 |
| <60 | | Ref. |  |  |
| 60-65 | | 1.88 (0.67 ; 5.25) | 0.229 |  |
| 66-70 | | 1.21 (0.44 ; 3.28) | 0.712 |  |
| >70 | | 1.89 (0.65 ; 5.49) | 0.243 |  |
| **Sex (men)** | | 0.98 (0.41 ; 2.31) |  | 0.956 |
| **BMI (Kg/m^2^)** | |  |  | **0.001** |
| <23.6 | | Ref. |  |  |
| 23.6-26.5 | | 2.54 (0.86 ; 7.48) | 0.090 |  |
| 26.6-29.3 | | **3.31 (1.07 ; 10.23)** | **0.038** |  |
| >29.3 | | **10.63 (3.23 ; 35.06)** | **<0.001** |  |
| **Waist/Hip ratio** | |  |  | 0.149 |
| <0.87 | | Ref. |  |  |
| 0.87-0.93 | | 2.28 (0.82 ; 6.35) | 0.115 |  |
| 0.94-0.99 | | 1.24 (0.43 ; 3.54) | 0.693 |  |
| >0.99 | | 3.43 (0.95 ; 12.33) | 0.059 |  |
| **Tobacco status** | |  |  | 0.730 |
| Never smoker | | Ref. |  |  |
| Former smoker | | 1.21 (0.53 ; 2.78) | 0.653 |  |
| Current smoker | | 0.79 (0.24 ; 2.60) | 0.703 |  |
| **Hypoglycaemic treatment** | | 5.56 (0.63 ; 48.92) |  | 0.122 |
| **Inhaled corticosteroid treatment** | | 3.40 (0.96 ; 12.08) |  | 0.058 |

Significant p-values and OR are shown in bold. HOMA-IR: Homeostasis model assessment of insulin resistance; COPD: chronic obstructive pulmonary disease; COPD2+: only GOLD stage 2 and 3 are compared with controls; BMI: body mass index. Ref.: reference category. Cox-Snell Model R^2^ = 0.30
