# Supplementary material for: Metabolic profiles among COPD and controls in the CanCOLD population-based cohort
Source: PLoS One. 2020 Apr 10;15(4):e0231072. doi: 10.1371/journal.pone.0231072 (PMC7147771; doi:10.1371/journal.pone.0231072)
Supplement: S7 Table — (DOCX) [file pone.0231072.s007.docx]

**Table S7** Multivariate logistic regression on Visceral adipose tissue cross-sectionnal area (VAT CSA) > 75^th^ percentile by sex of the total population

|  | **OR** **(95%CI)** | | **p - value** | |
| --- | --- | --- | --- | --- |
| **COPD** | | 0.83 (0.42 ; 1.64) |  | 0.596 |
| **Age** | |  |  | 0.754 |
| <60 years | | Ref. |  |  |
| 60-65 years | | 1.46 (0.57 ; 3.75) | 0.433 |  |
| 66-70 years | | 1.07 (0.41 ; 2.75) | 0.896 |  |
| >70 years | | 1.47 (0.60 ; 3.64) | 0.403 |  |
| **Tobacco status** | |  |  | 0.263 |
| Never smoker | | Ref. |  |  |
| Former smoker | | 1.94 (0.87 ; 4.35) | 0.108 |  |
| Current smoker | | 1.46 (0.53 ; 4.07) | 0.464 |  |
| **Hypoglycaemic treatment** | | **5.76 (1.85 ; 17.98)** |  | **0.003** |
| **Inhaled corticosteroid treatment** | | **3.15 (1.32 ; 7.51)** |  | **0.010** |
| **Hypolipemic treatment** | | **2.56 (1.28 ; 5.09)** |  | **0.008** |

Significant p-values and OR are shown in bold. COPD: chronic obstructive pulmonary disease; Ref.: reference category. Cox-Snell Model R^2^ = 0.14
