# Supplementary material for: Metabolic profiles among COPD and controls in the CanCOLD population-based cohort
Source: PLoS One. 2020 Apr 10;15(4):e0231072. doi: 10.1371/journal.pone.0231072 (PMC7147771; doi:10.1371/journal.pone.0231072)
Supplement: S8 Table — (DOCX) [file pone.0231072.s008.docx]

**Table S8** Multivariate logistic regression on Visceral adipose tissue cross-sectionnal area (VAT CSA) > 75^th^ percentile by sex of the total population, **COPD 2+ only**

|  | **OR** **(95%CI)** | | **p - value** | |
| --- | --- | --- | --- | --- |
| **COPD 2+** | | **2.27 (1.00 ; 5.15)** |  | **0.050** |
| **Age** | |  |  | 0.405 |
| <60 years | | Ref. |  |  |
| 60-65 years | | 1.68 (0.58 ; 4.86) | 0.336 |  |
| 66-70 years | | 1.04 (0.36 ; 3.03) | 0.942 |  |
| >70 years | | 2.06 (0.72 ; 5.88) | 0.175 |  |
| **Tobacco status** | |  |  | 0.293 |
| Never smoker | | Ref. |  |  |
| Former smoker | | 1.47 (0.61 ; 3.57) | 0.394 |  |
| Current smoker | | 0.69 (0.22 ; 2.19) | 0.523 |  |
| **Hypoglycaemic treatment** | | **11.42 (2.27 ; 57.43)** |  | **0.003** |
| **Inhaled corticosteroid treatment** | | 2.21 (0.78 ; 6.23) |  | 0.135 |
| **Hypolipemic treatment** | | **2.94 (1.33 ; 6.47)** |  | **0.008** |

Significant p-values and OR are shown in bold. COPD: chronic obstructive pulmonary disease; COPD2+: only GOLD stage 2 and 3 are compared with controls; Ref.: reference category. Cox-Snell Model R^2^ = 0.22
